# Modification of graphene oxide by angiopep-2 enhances anti-glioma efficiency of the nanoscaled delivery system for doxorubicin

**DOI:** 10.18632/aging.103275

**Published:** 2020-05-30

**Authors:** Yue Zhao, Hang Yin, Xiaoyu Zhang

**Affiliations:** 1 Radiotherapy Department, Cangzhou Central Hospital, Cangzhou 061000, China; 2 Department of Cardiology, Cangzhou People’s Hospital, Cangzhou 061000, China; 3 Department of Thyroid and Breast Surgery, Cangzhou Central Hospital, Cangzhou 061000, China

**Keywords:** angiopep-2, graphene oxide, doxorubicin, glioma

## Abstract

Objective: This study aimed to evaluate the efficacy of the improved nanoscaled delivery system for doxorubicin (Dox) based on angiopep (ANG)-2 modified graphene oxide (GO), the so-called ANG-Dox-GO, in suppressing the growth and and metastasis of glioma cells.

Results: Modification of GO by angiopep-2 significantly increased the cellular uptake of Dox. In addition, ANG-Dox-GO treatment of U87 MG cells significantly inhibited cell viability, decreased clone number, cell migration and invasion andinduced cell apoptosis, with superior efficiency over that of Dox-GO and free Dox. Similar results were observed in in vivo experiments—tumor size and weight of glioma xenograft mice were obviously decreased after treatments with ANG-Dox-GO, Dox-GO and Dox, respectively, as compared with control group, and the efficiency was the highest in ANG-Dox-GO, followed by Dox-Go and Dox.

Conclusions: ANG-Dox-GO exhibited superior anti-glioma effects over Dox-GO both in vitro and in vivo experiments.

Methods: The morphology of ANG-Dox-GO was analyzed by UV visible absorption spectroscopy and atomic force microscopy and its in vitro cellular uptake was measured using confocal imaging analysis. The antitumor effects of GO, unbound Dox, Dox-GO and ANG-Dox-GO were evaluated by MTT assay, colony-forming assay, cell apoptosis assay and Transwell assay in U87 malignant glioma (MG) cells.

## INTRODUCTION

Glioma is the most common form of primary malignant tumor that occurs in the central nervous system. It originates in the neurogenic ectoderm and is, characterized by high rates of metastasis, recurrence and mortality [1]. Currently, the conventional and effective therapeutic options for glioma are primarily sophisticated surgical resection, followed by chemoradiation and immunotherapy [2, 3]. Although rapid progress has been made in the diagnosis and treatments for glioma, poor prognosis and lower survival rate still pose threats to patients life [2]. Therefore, it is essential to uncover the underlying molecular mechanism of glioma as well as develop effective treatment options.

Currently, nanomaterials have shown bright promises in detection and treatment of cancers by exerting various targeted roles including imaging, immunodetection, chemotherapy, radiotherapy and immunotherapy [4]. In particular, graphene is a single-layer sheet-like and two-dimensional carbon atoms, which is characterized by sp2 hybridized hexagonal honeycomb structure [5]. Graphene oxide (GO), as an oxygenated derivative of graphene, contains a series of active oxygen-containing groups, and is usually prepared through treating graphene with a strong acid or a strong oxidant [6]. It has attracted tremendous attention due to its exceptional physicochemical properties, including good thermal stability, excellent mechanical strength and high electronic conductivity [6]. Recently, the biological applications of GO have been widely reported, including drug delivery, biomedicine, diagnostic tool for cancers and photothermography [7]. Several studies have proved anti-tumor effects of GO as a tumor targeting drug carrier [8–10].

Doxorubicin (Dox) is a commonly used antitumor drug that inhibits the synthesis of RNA and DNA [11]. However, its anti-tumor efficiency in clinical practice is compromised due to its low bioavailability and other side effects caused by nonspecific cytotoxicity [11]. Thus, increasing studies have designed an effective Dox-loaded drug delivery system to increase the intra-tumor concentration of Dox. Unfortunately, insufficient delivery across the blood brain barrier (BBB) remain a serious challenge for the treatment of gliomas. Notably, recent studies have demonstrated that angiopep-2, a peptide as a specific ligand for low-density lipoprotein receptor-related protein-1 (LRP-1), allowed for increased brain-targeting drug delivery due to its high transcytosis capacity and parenchymal accumulation in passing through the BBB [12–14]. In view of the above advantages, angiopep-2 modified nanocarriers loaded with Dox (ANG-Dox-GO) may offer an effective drug delivery system for the treatment of glioma.

In this study, the ANG-Dox-GO was prepared and characterized. Following that, the effects of ANG-Dox-GO on cellular uptake, cell proliferation, apoptosis and migration were evaluated in U87 MG cells. In addition, the anti-tumor effect of ANG-Dox-GO on glioma xenograft in nude mice was evaluated.

## RESULTS

### Characterization of ANG-Dox-GO

**Figure 1:**
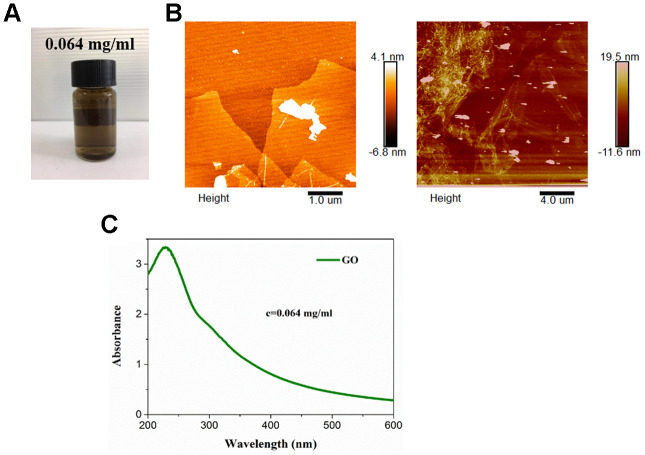
**Characterization of angiopep-2 polypeptide-modified and doxorubicin-loaded graphene oxide (ANG-Dox-GO).** (**A**) The appearance of commercial GO. (**B**) The representative images of ANG-Dox-GO using atomic force microscopy. (**C**) The ultraviolet visible absorption spectra of ANG-Dox-GO.

The color of commercial GO varies from yellow to brown (Figure 1A). Under a AFM, ANG-Dox-GO was 4-5 nm in thickness and 100-400 nm in diameter (Figure 1B), suggesting the formation of single or several layers of GO nanosheets. In addition, the optical absorption spectra of ANG-Dox-GO showed absorption peaks at approximately 230 nm (Figure 1C).

### Cellular uptake of ANG-Dox-GO

**Figure 2:**
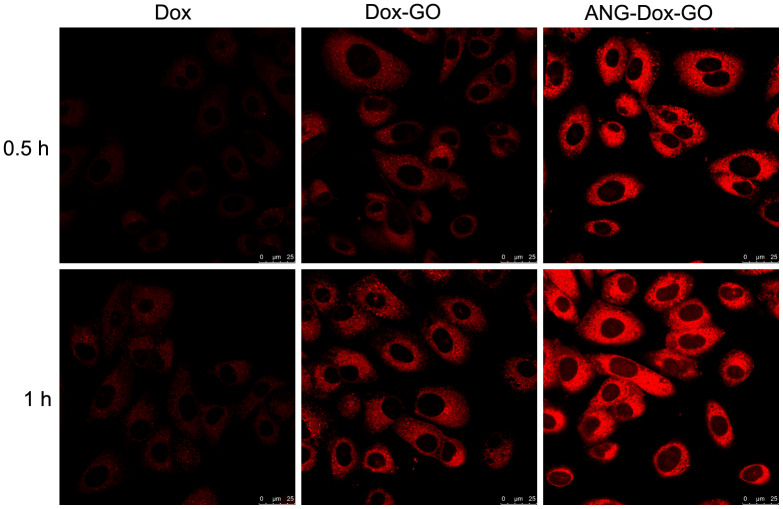
**Cellular uptake of Dox, doxorubicin-loaded graphene oxide (Dox-GO) or angiopep-2 polypeptide-modified and doxorubicin-loaded graphene oxide (ANG-Dox-GO) by U87 MG cells.** Confocal images of U87 MG cells treated with Dox, Dox-GO or ANG-Dox-GO at 10 μg/mL of Dox for 0.5 and 1 h.

Based on confocal imaging analysis, increased fluorescence intensity was detected in cells treated with ANG-Dox-GO, Dox-GO as compared with free Dox, and the strongest fluorescence intensity was found in cells treated with ANG-Dox-GO (Figure 2). These results indicated that the modification by angiopep-2 increased the cellular uptake of the delivery system. Moreover, the cellular uptake at 1 h of incubation time was higher than that at 0.5 h (Figure 2).

### Anti-proliferation effect of ANG-Dox-GO on U87 MG cells

**Figure 3:**
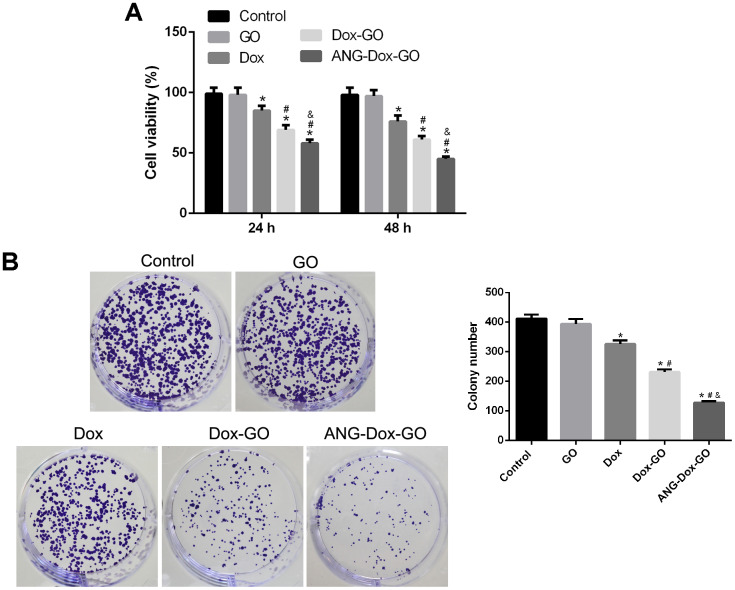
**Angiopep-2 polypeptide-modified and doxorubicin-loaded graphene oxide (ANG-Dox-GO) inhibits tumor growth in U87 MG cells.** (**A**) Cell viability of U87 MG cells treated with PBS (control), GO, Dox (30 μg/mL), Dox-GO (containing 30 μg/mL of Dox), and ANG-Dox-GO (containing 30 μg/mL of Dox), respectively, for 24 h and 48 h by MTT assay. (**B**) Clone number of U87 MG cells treated with PBS (control), GO, Dox (30 μg/mL), Dox-GO (containing 30 μg/mL of Dox), and ANG-Dox-GO (containing 30 μg/mL of Dox), respectively, for 24 h by colony formation assay. *P < 0.05 vs control cells; #P < 0.05 vs Dox; &P < 0.05 vs. Dox-GO.

Cell proliferation and apoptosis was utilized as the indicators to evaluate the antitumor effect of ANG-Dox-GO on U87 MG cells. Firstly, the cell viability was evaluated using MTT assay, and the results showed that compared with control cells, GO treatment exhibited few effects on cell viability in U87 MG cells; however, cell viability was significantly inhibited by Dox treatment (*p* < 0.05, Figure 3A) at 24 h and 48 h. In addition, compared with Dox treatment, U87 MG cells treated with Dox-GO exhibited lower cell viability, and ANG-Dox-GO treatment further inhibited cell viability (*p* < 0.05, Figure 3A). Similarly, colony formation assay also revealed that U87 MG cells treated with PBS had similar clone number compared with GO treatment, whereas the clone number was obviously reduced after free Dox treatment (*p* < 0.05, Figure 3B). Compared with cells treated with free Dox, the clone number was remarkably decreased in cells with Dox-GO, and cells treated with ANG-Dox-GO had reduced clone number compared with Dox-GO treatment (*p* < 0.05, Figure 3B).

### Pro-apoptosis effect of ANG-Dox-GO on U87 MG cells

**Figure 4:**
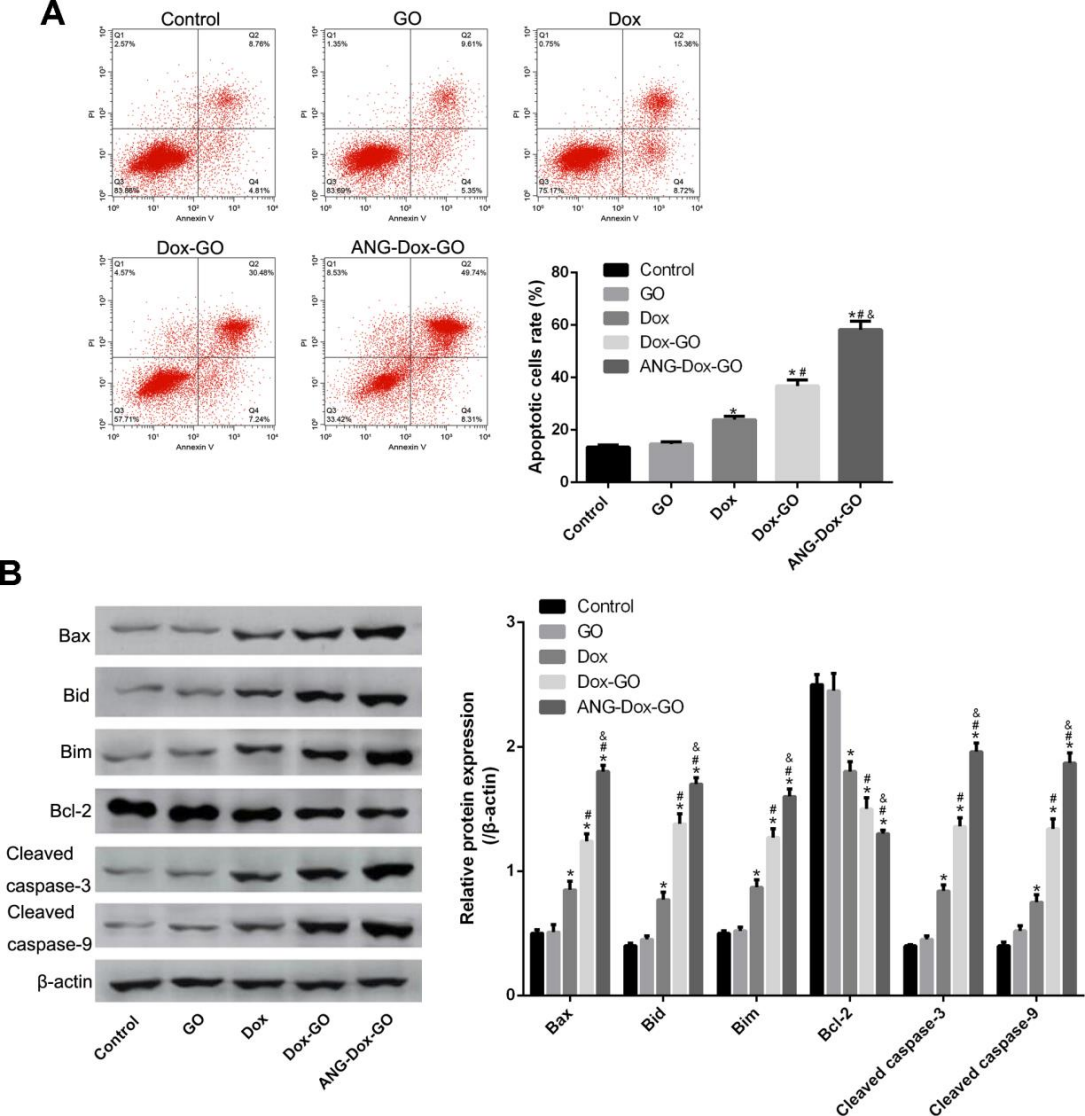
**Angiopep-2 polypeptide-modified and doxorubicin-loaded graphene oxide (ANG-Dox-GO) induces cell apoptosis in U87 MG cells.** (**A**) Cell apoptosis rate of U87 MG cells treated with PBS (control), GO, Dox (30 μg/mL), Dox-GO (containing 30 μg/mL of Dox), and ANG-Dox-GO (containing 30 μg/mL of Dox), respectively, for 24 h by flow cytometry analysis. (**B**) The expression of apoptosis-related proteins, including Bax, Bid, Bim, cleaved caspase-3, and cleaved caspase-9, in U87 MG cells treated with PBS (control), GO, Dox (30 μg/mL), Dox-GO (containing 30 μg/mL of Dox), and ANG-Dox-GO (containing 30 μg/mL of Dox), respectively, for 24 h by western blotting. *P < 0.05 vs control cells; #P < 0.05 vs Dox; &P < 0.05 vs. Dox-GO.

Flow cytometry analysis revealed that the apoptotic rate (AR) of cells treated with free Dox was significantly increased as compared with control cells or cells treated with GO; meanwhile, compared with U87 MG cells treated with free Dox, cells treated with Dox-GO had increased AR, and the AR was further decreased in cells treated with ANG-Dox-GO (*p* < 0.05, Figure 4A). Meanwhile, the expression of apoptosis-related proteins, including Bax, Bid, Bim, Bcl-2, cleaved caspase-3 and cleaved caspase-9, was detected using western blotting analysis. The results indicated that compared with control cells or cells treated with GO, cells treated with free Dox had remarkably increased protein levels of Bax, Bid, Bim, cleaved caspase-3, and cleaved caspase-9, declined protein level of Bcl-2 (*p* < 0.05, Figure 4B). Moreover, when compared to cells treated with free Dox, cells treated with Dox-GO or ANG-Dox-GO had higher protein levels of Bax, Bid, Bim, cleaved caspase-3 and cleaved caspase-9 and declined protein level of Bcl-2, especially the cells treated with ANG-Dox-GO (*p* < 0.05, Figure 4B).

### Anti-migration effect of ANG-Dox-GO on U87 MG cells

**Figure 5:**
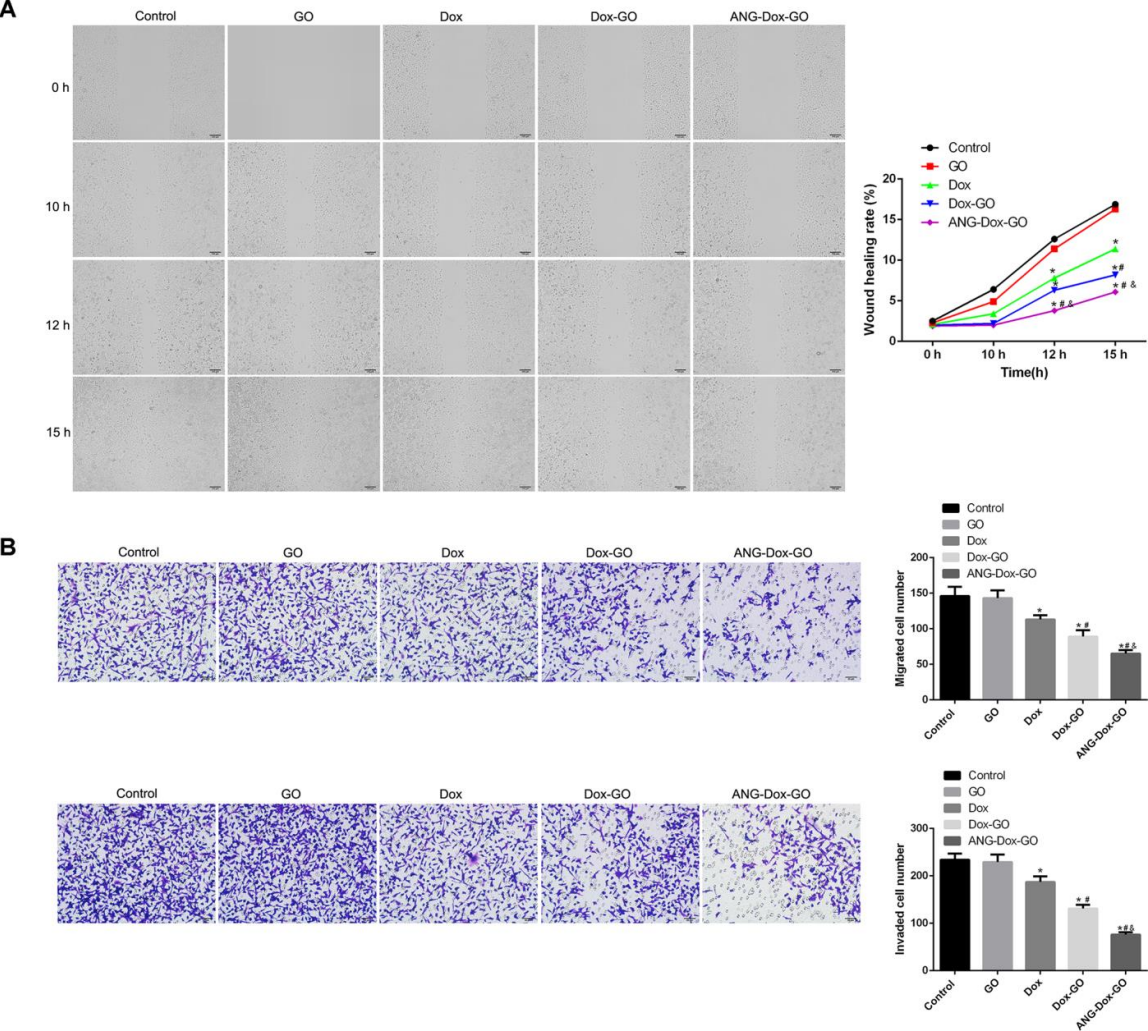
**Angiopep-2 polypeptide-modified and doxorubicin-loaded graphene oxide (ANG-Dox-GO) inhibits metastasis in U87 MG cells.** (**A**) The wound healing rate of U87 MG cells treated with PBS (control), GO, Dox (30 μg/mL), Dox-GO (containing 30 μg/mL of Dox), and ANG-Dox-GO (containing 30 μg/mL of Dox), respectively, at 0, 10, 12 h and 15 h by wound healing assay. (**B**) Cell migration and migration rates in U87 MG cells treated with PBS (control), GO, Dox (30 μg/mL), Dox-GO (containing 30 μg/mL of Dox), and ANG-Dox-GO (containing 30 μg/mL of Dox), respectively, by Transwell assay. *P < 0.05 vs control cells; #P < 0.05 vs Dox; &P < 0.05 vs. Dox-GO.

Wound healing assay showed that GO exhibited no effect on wound healing in U87 MG cells compared with control cells, while free Dox significantly inhibited the wound healing rate of U87 MG cells in time-dependent manner (*p* < 0.05, Figure 5A). In addition, compared with free Dox treatment, Dox-GO treatment was associated with decreased wound healing rate, and ANG-Dox-GO treatment further decreased the wound healing rate (*p* < 0.05, Figure 5A). Similarly, Transwell assay also revealed that cell migration and invasion were dramatically inhibited in U87 MG cells treated with free Dox compared with control cells or cells treated with GO (*p* < 0.05, Figure 3B). The rates of cell migration and invasion were remarkably decreased in cells treated with Dox-GO as compared with cells treated with free Dox, and cells treated with ANG-Dox-GO showed lower rates of cell migration and invasion than those treated with Dox-GO (*p* < 0.05, Figure 5B).

### Effect of ANG-Dox-GO on glioma xenograft in nude mice

**Figure 6:**
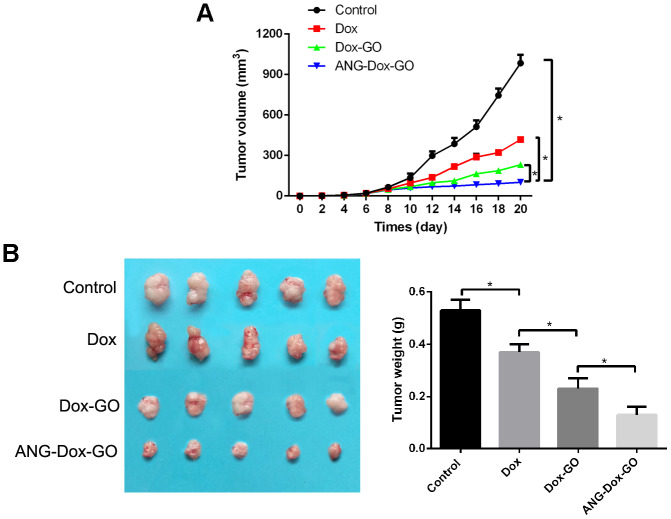
**Angiopep-2 polypeptide-modified and doxorubicin-loaded graphene oxide (ANG-Dox-GO) exhibited better anti-tumor effect of glioma xenograft mice.** (**A**) The tumor volumes of mice with different treatments, including PBS (control), Dox (2 mg/kg), Dox-GO (containing 2 mg/kg of Dox), and ANG-Dox-GO (containing 2 mg/kg of Dox), respectively, every two days for 20 days. (**B**) The tumor weights of mice with different treatments, including PBS (control), Dox (2 mg/kg), Dox-GO (containing 2 mg/kg of Dox), and ANG-Dox-GO (containing 2 mg/kg of Dox), respectively, on 20 days. *P < 0.05.

In vivo experiments showed that tumor size of mice xenografted with human glioma cells was decreased by Dox treatment compared with that of the control group in a time-dependent manner, and the tumor size was further reduced in Dox-GO and ANG-Dox-GO treated groups, especially in ANG-Dox-GO group (*p* < 0.05, Figure 6A). Moreover, compared to control groups, tumor weight of mice was the lowest in the ANG-Dox-GO group, followed by Dox-GO and Dox treated groups (*p* < 0.05, Figure 6B), indicating superior anti-tumor effect of ANG-Dox-GO on glioma xenograft in nude mice.

## DISCUSSION

In this study, Dox-GO and ANG-Dox-GO were successfully prepared. And it was found that angiopep-2 modification significantly increased the cellular uptake of Dox. In addition, compared with control cells, cell treated with GO had little-to-no effects on cell proliferation, apoptosis and migration in U87 MG cells; however, ANG-Dox-GO treatment significantly inhibited cell viability, decreased clone number, suppressed cell migration and invasion, as well as induced cell apoptosis, the efficiency was followed by Dox-GO and free Dox treatment. Similarly, in vivo experiments revealed that compared with control group, tumor size and weight of mice xenografted with human glioma were obviously decreased after Dox treatment and further reduced in Dox-GO and ANG-Dox-GO treatment groups, especially in ANG-Dox-GO.

As one of the most commonly used chemotherapeutic drugs, Dox has been indicated for acute leukemia, malignant lymphoma, glioma, lung cancer, bladder cancer and other malignant tumors [11]. In this study, free Dox was found to have significantly inhibited cell viability, decreased clone number, suppressed migration and invasion, as well as increased the AR in U87 MG cells. Moreover, the protein levels of apoptosis-related proteins, including Bim, Bad, Bax, Bcl-2, cleaved caspase-3 and cleaved caspase-9, were measured. Bim, Bad, Bax and Bcl-2 are the members of Bcl-2 protein family, which play significant roles in cell apoptosis; to be specific Bim, Bad and Bax are pro-apoptosis proteins, while Bcl-2 is characterized as an anti-apoptosis protein [15]. Bcl-2 protein family has been found to be closely related with the signaling pathway of mitochondria-mediated apoptosis [16]. Caspase-3, a downstream molecule of apoptosis pathway, downregulates the ratio of Bcl-2 and Bax protein expression hence contributing to cell apoptosis [16]. In addition, caspase-3 can also be activated by recruitment and activation of caspase-9 [17]. Furthermore, our in vivo experiments also suggested the anti-tumor effect of Dox on glioma, which were supported by previous clinic trials [18, 19].

However, due to poor water solubility, clinical application of many hydrophobic anti-tumor drugs has been limited. GO was an exception. Given its highly hydrophilic nature and superior biocompatibility that excluding the inner defects of hydrophobic drugs, GO has been reported to be a promising nanocarrier without the [20, 21]. Dorniani D et al. [22] have demonstrated that gallic acid-loaded GO could improve the release of gallic acid and inhibit growth of liver cancer cells instead of normal fibroblasts. Another in vitro study has also shown that Dox loaded GO significantly inhibited cell proliferation and induced apoptosis as compared with free Dox in drug-resistant breast cancer cells [23]. In this study, Dox, a hydrophobic anti-cancer drug, was loaded into GO to improve drug delivery efficiency. Consistent with previous studies, this study also revealed that compared with free Dox, Dox-GO and ANG-Dox-GO treatment significantly diminished cell proliferation, migration and invasion, and induced cell apoptosis in U87 MG cells as well as enhanced anti-tumor effect on glioma xenograft in nude mice. These results confirmed the preferable anti-tumor effects of Dox-GO and ANG-Dox-GO than free Dox in vitro and in vivo.

Furthermore, due to poor permeability of chemotherapeutic drugs across the BBB, we particularly investigated the anti-tumor effects of angiopep-2 modified nanocarrier. LRP-1 has been found to be overexpressed on the BBB and participate in the transcytosis of various ligands such as melanotransferrin and lactoferrin across the BBB [24, 25]. Moreover, the overexpression of LRP-1 has also been detected in glioma cells, indicating that targeting LRP-1 may be a potential treatment option for glioma targeted drug delivery [26]. Notably, increasing evidence has demonstrated that angiopep-2 modified drug delivery system can enhance brain penetration of drugs across the BBB by targeting LRP-1 [27–29]. Consistently, this study proved that ANG-Dox-GO treatment further inhibited cell growth and metastasis in vitro and in vivo as compared with other Dox-GO nanocarriers. These results indicated that ANG-Dox-GO boasted enhanced anti-tumor effects than Dox-GO and free Dox through increasing brain penetration of Dox.

## MATERIALS AND METHODS

### Preparation and characterization of ANG-Dox-GO

GO was purchased from Jiangsu XFNANO Materials Tech Co. Ltd (Nanjing, Jiangsu, China). In brief, 3 mg of GO was added into an Erlenmeyer flask containing 20 mL of Dox solution (40 μg/L), and then the flask were shaken at 130 rpm for 160 min in 30°C water bath. Afterwards, the mixture was adjusted to a corresponding pH value with 0.01-1.0 mol/L of hydrochloric acid and sodium hydroxide solution. Next, the supernatant was obtained using a 0.45 μm aqueous phase filter. Subsequently, Dox-GO was modified by angiopep-2 through covalent bonding of amino and carboxyl groups to obtain angiopep-2 polypeptide-modified GO drug delivery system (ANG-Dox-GO).

The ultraviolet (UV) visible spectra of ANG-Dox-GO was measured by UV visible absorption spectroscopy (Biochrom, Cambridge, UK). The morphology and thickness of ANG-Dox-GO were evaluated by atomic force microscopy (AFM, JPK Instruments AG, Berlin, Germany).

### Cell culture and treatment

Human glioma cell line U87 MG was obtained from Shanghai Obio Technology Co., Ltd., and incubated in complete DMEM medium (Gibco, Carlsbad, CA, USA) at 37°C and in a 5% CO_2_ incubator. To evaluate the anti-tumor effects of ANG-Dox-GO on U87 MG cells, the cells were exposed to PBS (control), GO, Dox (30 μg/mL), Dox-GO (containing 30 μg/mL of Dox) and ANG-Dox-GO (containing 30 μg/mL of Dox), respectively.

### Observation of cell uptake

Cellular uptake of ANG-Dox-GO was evaluated using confocal imaging analysis. Briefly, U87 MG cells were seeded at the density of 2.5 × 10^4^ per well to 24-well plates for 24 h and incubated in the presence of Dox (10 μg/mL), Dox-GO (containing 10 μg/mL of Dox) and ANG-Dox-GO (containing 10 μg/mL of Dox) for 0.5h and 1 h, respectively. After being rinsed with PBS, the cells were observed using confocal fluorescence microscope with an argon-ion laser (488 nm).

### MTT assay

U87 MG cells were grown on 96-well plates, and received the above treatments. After conventional incubation for 24 and 48 h, each well was added with 10 μl of MTT (5 mg/mL, Sigma) to incubate for another 4 h. And 100 μL of dimethyl sulfoxide was added. Finally, cell viability was evaluated using a microplate spectrophotometer to measure absorbance at 470 nm.

### Colony-forming assay

U87 MG cells were seeded to 6-well plates at a density of 400 cells per well, and treated in the same way as described above for 14 days under standard culture conditions. Then the cells were fixed with absolute methanol, and incubated with crystal violet. Ultimately, the number of colonies was counted.

### Cell apoptosis assay

FITC-Annexin V Apoptosis kit was used. U87 MG cells received the above treatments for 24 h. Trypsin was added to digest U87 MG cells, and then the cells were harvested. Next, cells were rinsed with PBS and re-suspended in binding buffer. After the cells were incubated in the presence of FITC-Annexin V and PI for 15 min, the number of apoptotic cells was calculated using a flow cytometer (BD, CA, USA).

### Wound healing assay

U87 MG cells were inoculated in 6-well plates. After growing to 60% confluence, cells were wounded by scratching with sterile plastic pipette tips vertically against the well, followed by incubation only in DMEM without serum. Subsequently, cells were treated with PBS (control), GO, Dox (30 μg/mL), Dox-GO (containing 30 μg/mL of Dox) and ANG-Dox-GO (containing 30 μg/mL of Dox), respectively. The distance of the scratch was measured at 0, 10, 12 and 15 h using a light microscope (Olympus, Japan).

### Transwell assay

Tumor cell invasion and migration were elevated by Transwell chambers (Corning). To be specific, the lower compartment was filled with DMEM supplemented with 10% FBS. The U87 MG cells with different treatments were cultured in the upper compartment coated with Matrigel Matrix in medium free of serum for 24 h. Subsequently, the cells in the lower compartment was fixed and stained with 4,6-diamidino-2-phenylindole. And cell migration and invasion were evaluated using an inverted microscope (Olympus, Japan).

### Western blotting analysis

U87 MG cells were treated for 24 h with PBS (control), GO, Dox (30 μg/mL), Dox-GO (containing 30 μg/mL of Dox) and ANG-Dox-GO (containing 30 μg/mL of Dox), respectively. The harvested cells were lysed by RIPA lysis buffer (Gibco), and then proteins were extracted with a commercial kit (Pierce, Rockford, IL, USA). The extracted protein was resolved on PAGE gel, and the protein sample was transferred to PVDF membrane, which was blocked and reacted with Bax, Bid, Bim, Bcl-2, cleaved caspase-3, cleaved caspase-9 or β-actin primary antibody (1:300, Santa Cruz) overnight at 4°C, respectively.. After incubation in the presence of the second antibody (1:5000, Jackson, USA), the protein levels were determined using enhanced chemiluminescence (ECL, Millipore, USA).

### Animal model and treatments

The experiments were approved by the local Animal Ethics Committee of Cangzhou Central Hospital prior to initiation. Healthy nude mice, weighting 18-22 g, purchased from Charles River, Beijing, China, were used for the following experiments after one week of acclimation. To construct the xenografted mouse model, 1×10^6^ of U87 MG cells were inoculated subcutaneously in each mouse. Then, xenografted mice were treated with PBS (control), Dox (2 mg/kg), Dox-GO (containing 2 mg/kg of Dox) and ANG-Dox-GO (containing 2 mg/kg of Dox) by rapid tail vein injection, respectively. The tumor size of each mouse was monitored every two days for 20 days. On day 20, mice were euthanized, and the tumor was resected for measurement of tumor weight.

### Statistical analysis

Data was presented as mean ± SD. Data was compared using one-way ANOVA followed by multiple comparison tests based on SPSS software. *P* < 0.05 was considered statistically significant.
